# The nasal lymphatic route of CSF outflow: implications for neurodegenerative disease diagnosis and monitoring

**DOI:** 10.1080/19768354.2024.2307559

**Published:** 2024-01-29

**Authors:** Jiwon Chae, Mina Choi, Juyoung Choi, Seung-Jun Yoo

**Affiliations:** a Department of Life Science, College of Natural Sciences, Hanyang University, Seoul, Republic of Korea; b Keybasic Co., ltd, Seoul, Republic of Korea; c Research Institute for Natural Sciences, Hanyang University, Seoul, Republic of Korea; d Research Institute for Convergence of Basic Sciences, Hanyang University, Seoul, Republic of Korea; e Hanyang Institute of Bioscience and Biotechnology, Hanyang University, Seoul, Republic of Korea

**Keywords:** Cerebrospinal fluid, brain lymphatics, glymphatic system, cribriform plate, neurodegenerative diseases

## Abstract

Cerebrospinal fluid (CSF) plays a crucial role in the brain's lymphatics as it traverses the central nervous system (CNS). Its primary function is to facilitate the outward transport of waste. Among the various CSF outflow pathways, the route through the cribriform plate along the olfactory nerves stands out as the most predominant. This review describes the outflow pathway of CSF into the nasal lymphatics. Additionally, we examine existing studies to describe mutual influences observed between the brain and extracranial regions due to this outflow pathway. Notably, pathological conditions in the CNS often influence CSF outflow, leading to observable changes in extracranial regions. The established connection between the brain and the nose is significant, and our review underscores its potential relevance in monitoring CNS ailments, including neurodegenerative diseases. Considering that aging – the most significant risk factor for the onset of neurodegeneration – is also a principal factor in CSF turnover alterations, we suggest a novel approach to studying neurodegenerative diseases in therapeutic terms.

## Introduction

Cerebrospinal fluid (CSF) is a fluid that flows within the central nervous system (CNS), and most is found in the cranial or spinal subarachnoid space (SAS). As it circulates within the SAS, CSF contributes to maintaining fluid homeostasis and buoyancy within the CNS (Proulx 2021). CSF is generally secreted daily in the choroid plexuses, and it circulates to sites of absorption. It is then either reabsorbed through pathways connected to the venous outflow system or drained through specific lymphatic outflow pathways, participating in the brain lymphatics (Sakka et al. 2011). The glymphatic system of the brain facilitates CSF outflow to eliminate unwanted metabolites from the CNS, including pathogenic proteins of neuropathy (Persson et al. 2022).

One of the representative paths that CSF drains from the brain parenchyma is through the cribriform plate, and this path flows along the olfactory nerves. Histochemistry and advanced microscopy have revealed the structures of lymphatic vessels and their relations with nerves in the nasal area, where lymphatics are found either in dura mater above the cribriform plate and under the nasal mucosa, providing a CSF drainage route along the olfactory nerves (Furukawa et al. 2008). As CSF exits the brain, the majority of it traverses the cribriform plate, and is absorbed into the lymphatics situated in the submucosa of the olfactory epithelium (Nagra et al. 2006). With its direct connection with SAS, nasal submucosal lymphatic vessels can drain CSF from intracranial (Spera et al. 2023). This CSF outflow path provides a physical connection between the nasal lymphatics, olfactory system, and brain. Furthermore, the CSF flow from one space to another provides evidence of the relationship between the brain and extracranial regions, as shown in previous studies. The interaction between the brain and extracranial regions mediated by CSF is primarily associated with brain immunity. Macromolecules and immune cells travel through the olfactory nerve sheaths, passing through the cribriform plate into the nasal cavity when CSF is drained from the SAS. From there, they can reach the deep cervical lymph nodes through the Nasal-associated lymphoid tissue (NALT) (Aspelund et al. 2015; Louveau et al. 2015; Engelhardt et al. 2017). Furthermore, the exchange of fluids bounded by the cribriform plate might not be unidirectional, supported by the fact that intranasally injected aqueous solutions can access the olfactory bulb, meninges, and SAS through the same vascular structures (Faber 1937). A more comprehensive examination of the conditions resulting from these relationships will be addressed in the subsequent section: Connection between the brain and extracranial region through the CSF outflow path into the nasal lymphatics (Figure 2). This link suggests that changes observed in the CSF may be reflected in extracranial areas, including the nasal lymphatics and olfactory system.

It has been established that there is a CSF outflow pathway through and an associated relationship with the nasal lymphatics, which suggests that changes in CSF flow in neurodegenerative disease conditions may influence the olfactory system. Certain changes in olfactory features have been observed in various neurodegenerative diseases. In various neurological diseases – such as Alzheimer's disease, Parkinson's disease, multiple sclerosis, schizophrenia, and depression – decline in olfaction ability is identified in their early stage, prior to onset of cognitive or motor symptoms. For instance, olfactory dysfunction has been identified in 90% of PD patients and 85% of AD patients in the early stage (Hu et al. 2015; Dan et al. 2021). The fact that olfactory dysfunction usually precedes other typical symptoms of neurological diseases proposes that the olfactory system might have a high vulnerability in such diseases (Kim et al. 2018). Therefore, understanding this outflow pathway could provide new perspectives for the diagnosis and monitoring of neurodegenerative diseases.

## CSF dynamics and outflow pathways in the mammalian brain

In the mammalian brains, CSF is predominantly present in the SAS surrounding the exterior of the brain and is also found in the ventricles within the interior of the brain. As a CSF-filled space with blood vessels and cisterns, the SAS is located between the arachnoid mater and pia mater, enveloping the CNS. CSF is mainly produced in the choroid plexus in the ventricles of the brain, and it is recycled with a cycle of approximately 26 h (Johanson et al. 2008). Newly produced CSF flows within the SAS and the ventricles, and its flow is maintained by arterial pulsations and respiratory movements. CSF serves several functions including providing buoyancy, acting as a protective layer that can absorb physical shocks, facilitating the transmission of chemical signals and removing waste materials to maintain the CNS (Simon and Iliff 2016).

After circulating throughout the CNS, CSF is either reabsorbed by the venous system or drained into the lymphatics. The gradual change in intracranial pressure (ICP) regulates transport of CSF through the arachnoid villi route, and the CSF flow distribution into each different drainage pathway is dependent on ICP (Boulton et al. 1998; Vinje et al. 2020). Arachnoid granulations, which are protrusions of the arachnoid mater that extend outward in the direction of the outer membrane of the dura mater, act as the main contributor to CSF outflow by allowing CSF to move from the SAS to the bloodstream through the dural venous sinuses. CSF also drains from the brain via subarachnoid sleeves, which lead to peripheral lymphatic vessels (Rasmussen et al. 2020).

## CSF drainage maintains the ‘brain lymphatic system’

Despite its relatively high metabolic rate, the brain lacks a conventional lymphatic circulation. Consequently, the brain necessitates alternative mechanisms for waste clearance in order to maintain its immunity and eliminate unwanted products (Iliff et al. 2012). The presence of the glymphatic system and meningeal lymphatics engages in the exchange and transport of soluble metabolites in the brain, thus maintaining the fluid dynamics of CNS (Yankova et al. 2021).

Glymphatic system is an analog of lymphatic system in CNS, as it contributes to its fluid homeostasis and neuroinflammation (Hablitz and Nedergaard 2021). The primary purpose of the glymphatic system is to facilitate the extravascular transport of hydrophilic solutes that are impermeable to brain–blood barrier (Hladky and Barrand 2022). It enables waste clearance through the flow of CSF within the SAS and arterial perivascular space, as a glial cell-dependent system of perivascular channels located within the brain. Astrocytes, equipped with the water channel aquaporin-4 (AQP4) in their endfeet, encircle the perivascular tunnels within the glymphatic system and support the fluid transport (Hablitz et al. 2020). The solutes that are targeted for removal via the glymphatic pathway encompass misfolded proteins originating from neurological defects, and these solutes ultimately traverse the meningeal lymphatics to reach the cervical lymphatic vessels (Louveau et al. 2017; Li et al. 2022).

The function of the glymphatic system is primarily influenced by factors such as the impact of sympathetic/parasympathetic innervation and the sleep-wake cycle. Furthermore, age-related changes in the brain can impede meningeal lymphatic drainage, resulting in functional impairment of the glymphatic pathway of the brain. Glymphatic pathway dysfunction can lead to the accumulation of toxic misfolded proteins, ultimately culminating in prolonged inflammation (Nycz and Mandera 2021). Any pathology that arises in the brain, including neurodegenerative diseases, has the capacity to disrupt the proper functioning of the glymphatic pathway (Sun et al. 2018).

Meningeal membranes surround the CNS, consisting of dura mater and leptomeninges – which is consisted of arachnoid mater and pia mater, forming the boundary of the SAS (Weller et al. 2018). Meningeal lymphatic vessels (MLVs), which are found in certain regions of the dura mater within the CNS, integrate the cerebrovascular and periventricular routes (Noé and Marchi 2019). In the olfactory system, the meningeal lymphatic network lies beneath the olfactory bulb, adjacent to the cribriform plate (Hsu et al. 2021). MLVs contribute to the fluid dynamics of the brain parenchyma by participating in the circulation of fluid within the brain and facilitating the exchange of soluble contents between cerebrospinal fluid and interstitial fluid. Also, Meningeal lymphatics are linked to the paravascular activity of CSF and interstitial fluid (ISF), allowing them to regulate the influx of CSF-borne immune neuromodulators into the brain and exert consequential effects (Da Mesquita et al. 2018a). They provide a drainage pathway for CSF towards the peripheral blood and offer a route for the removal of macromolecules, facilitating the elimination of waste products in the brain (Tamura et al. 2020; Jacob et al. 2022).

The integrity of the brain lymphatic system relies on the uninterrupted circulation of CSF and, notably, its efficient drainage. Consequently, the optimal functioning of CSF drainage plays a critical role in preserving neural function within the brain lymphatics and facilitating the removal of CSF-borne metabolic products (Brady et al. 2020).

## Predominance of the CSF outflow path through the cribriform plate

**Figure 1.**
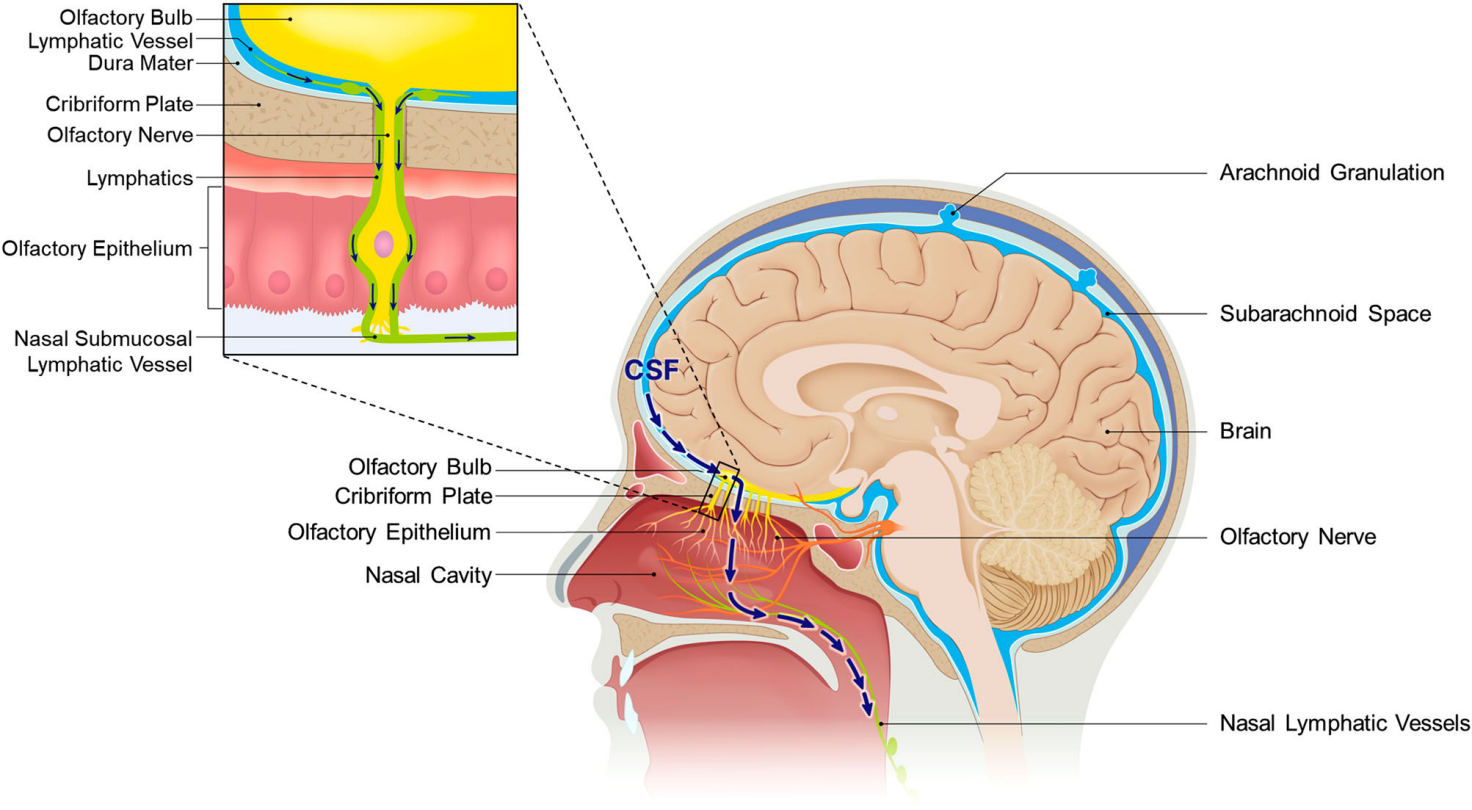
Sagittal view of CSF outflow through the cribriform plate. The arrow depicts CSF outflow path into nasal lymphatics. When CSF is drained towards the nasal, the lymphatics responsible for draining CSF surround the olfactory nerves and traverse the cribriform plate along with them. Through the connections established with nasal lymphatics and lymphatics adjacent to the cribriform plate, they can reach the nasal submucosa lining the nasal cavity. Ultimately, they are directed to cervical lymphatics via the lymphatic network.

As it exits the brain through the lymphatic pathway, CSF can flow alongside the cranial nerves, and a substantial portion travels through the olfactory sensory nerves and reaches the nasal mucosa (Figure 1). During this course, CSF traverses the cribriform plate of the skull, exiting the brain and moving into the external environment (Liu et al. 2012).

The existence and underlying principles of an outflow pathway that traverses the cribriform plate have been substantiated by various structural characteristics. Specifically, within the cribriform plate, lymphatic vessels situated near the brain have been observed to pass through it and establish connections directed towards the extracranial region (Hsu et al. 2019). The adjacent region of the arachnoid barrier exhibits a discontinuous structure that allows for the access of bulk flow of CSF. Within this context, the lymphatic vessels passing through the SAS and cribriform plate form a directly connected structure that envelops the surrounding olfactory nerves. The lymphatic vessels adjacent to the olfactory bulbs, which constitute the olfactory system, create a functionally continuous lymphatic network with the nasal mucosa that extends all the way to the cervical lymph nodes (Spera et al. 2023). Consequently, in this pathway, CSF previously residing within the SAS can pass through the foramina of the cribriform plate alongside the olfactory nerves, reaching the nasal lymphatics and ultimately the cervical lymph nodes. This route toward the nasal lymphatics constitutes a significant portion of the lymphatic outflow pathway of CSF. Various studies have proven the existence of this pathway conclusively, utilizing techniques such as tracer tracking, and have clearly established it as the major route for CSF drainage. For instance, Johnston et al. showed that when they injected yellow microfil into the CSF compartment of humans and other mammals, and observed at least 7 h later or after sacrifice, the microfils were predominantly distributed in the olfactory bulbs and the SAS adjacent to the cribriform plate (Johnston et al. 2004). Additionally, in tracer experiments involving the injection of [125I] albumin into the ventricles of rodents, a significant proportion of the injected amount was confirmed to be present in the olfactory turbinates, confirming the same results. Particularly, in the experiments by Bradbury and Westrop, when the cribriform plate was blocked during injection, recovery rate in cannulated jugular lymphatic trunk decreased in certain rate, indicating a substantial contribution of CSF drainage route through the cribriform plate in the connection between CNS and cervical lymphatics (Bradbury and Westrop 1983; Nagra et al. 2006). Other previous studies using other tracers – such as Kida et al.'s study using Indian Ink, have also examined the predominance of the nasal route in CSF drainage (Kida et al. 1993).

## Connection between the brain and extracranial region through the CSF outflow path into the nasal lymphatics

The presence of a CSF outflow path through the cribriform plate provides a physical and tangible connection between the brain and the nose. For instance, diseases such as CSF rhinorrhoea, in which CSF leaks through the nose, strongly support the existence of this physical link. Furthermore, the drainage of CSF through the cribriform plate – indicating the physical communication between the brain and the nose – allows for the possibility of interconnected changes, where an event on one side can potentially impact the other side. This can be observed through specific cases that confirm the presence of such interconnected changes across the cribriform plate boundary (Mehta et al. 2022).

**Figure 2.**
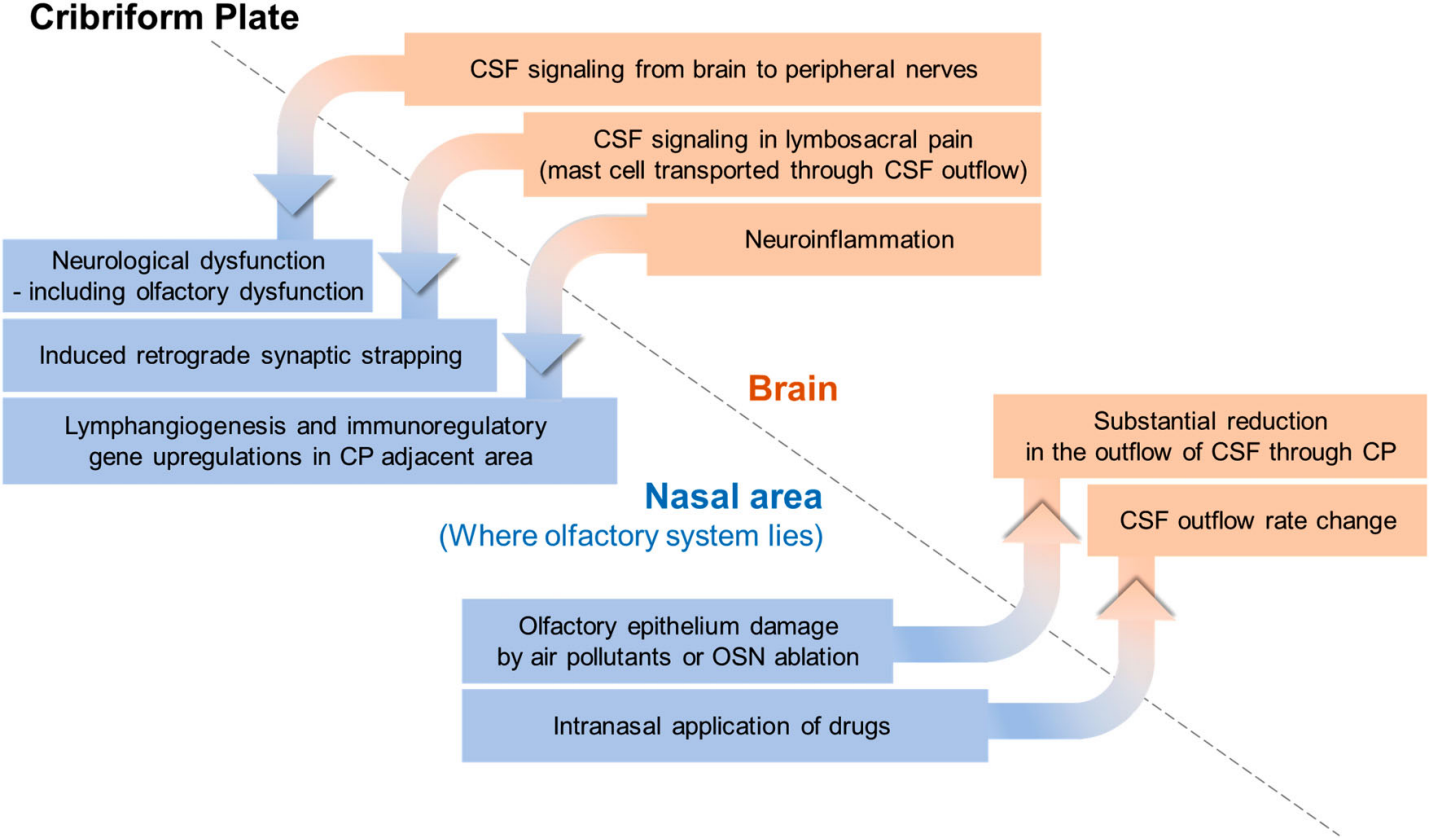
Interconnected changes found in brain and nasal area, bounded by the cribriform plate.

Changes in the nasal environment can induce alterations in CSF turnover, which in turn can impact the brain. The nasal mucosa, which is where most of the olfactory system is located, is an organ that is directly exposed to the external environment. Consequently, environmental factors such as air pollutants or drugs that change in the external environment can directly affect the olfactory region, including the olfactory sensory neurons, located near the nasal mucosa. There have been documented cases in which such influences have been found to directly affect CSF flow (Figure 2). When the olfactory epithelium is damaged by air pollutants or through chemical ablation of olfactory sensory neurons, substantial reduction in the outflow of CSF through the cribriform plate can occur (Norwood et al. 2019). Furthermore, intranasal administration of drugs has been found to affect the outflow of CSF, establishing a link between the nasal cavity and the brain. For instance, prostaglandin analogs applied to the nasal mucosa through nasal inhalation have been demonstrated to increase CSF outflow (Pedler et al. 2021). These discoveries highlight the regulatory potential of intranasal delivery of external substances in modulating CSF outflow, potentially influencing lymphatic contractile activity. In addition, when delivered intranasally,,nebulized pharmacological agents have been found to hold promise for non-invasive regulation of CSF absorption and outflow resistance (Kim et al. 2014).

This correlation can be observed in the reverse direction, extending from the brain to the extracranial region beyond the cribriform plate (Figure 2). It has been demonstrated that CSF not only serves as a drainage fluid but can also provide a route for signal transmission within the brain, linking the intracerebral process with the outer environment (Illes 2017). Various pieces of evidence, including anatomical features, relatively high signals detected in CSF than plasma, and correlations between CSF levels and behaviors such as sex behavior and CSF-GnRH level, support the presence of CSF signaling (Lehman and Silver 2000). Facilitated by the CSF outflow pathway, CSF signaling has been shown to extend along both the brain and peripheral nerves, to reach peripheral tissues. CSF signaling has been associated with the occurrence and progression of neurological dysfunctions, including dysautonomia and olfactory dysfunction (Bechter 2013). Moreover, in the context of lumbosacral pain, substances released by mast cells can be transported peripherally through the CSF outflow pathway, potentially inducing consequences such as retrograde synaptic strapping in the nerves, mediated by CSF signaling (Bechter and Schmitz 2014).

Under neuroinflammatory conditions, meningeal lymphatics in proximity to the cribriform plate undergo lymphangiogenesis, leading to the formation of lymphatic vessels with altered phenotypes, facilitated by the upregulation of immunoregulatory genes (Hsu et al. 2022).

These features suggest that the CSF outflow route through the cribriform plate not only serves as a physical pathway but also exhibits a reciprocal correlation, where changes on one side can be reflected on the other side.

## Age-related alterations in CSF outflow pathways

As environmental factors that influence CSF outflow, age-related changes can result in critical alterations in CSF. ICP serves as a key regulator of CSF flow. Notably, ICP is significantly influenced by the normal aging process, and alterations in ICP can have a critical impact on the overall turnover of CSF (Pedersen et al. 2018).

Aging induces changes in the distribution of CSF elimination routes. In a mouse study, older mice showed a decreased relative contribution of the nasal route to CSF outflow compared with younger mice; however the outflow through the spinal route did not exhibit the same trend (Ma et al. 2017; Brady et al. 2020).

Moreover, aging is linked to impaired glymphatic function, and the alterations observed in the glymphatic-lymphatic clearance process have been demonstrated to contribute to the accumulation of pathological proteins in models of neurodegenerative diseases (Lee et al. 2020b). Consequential alterations in the aging brain, including those that affect CSF drainage, show similarities to changes observed in patients with neurodegenerative disease. Age is considered the primary risk factor for the onset of neurodegenerative diseases, emphasizing the connection between aging and these conditions.

There are also major age-related alterations in terms of general lymphatics, both functionally and structurally. Lymphatic collector functions, such as contractile pressure and pumping frequency, are noted to decrease with age, while lymphatic permeability tends to increase with age (Jakic et al. 2020). The loss of endothelial glycocalyx contributes to increased permeability, and reduced coverage of muscle cells diminishes their contractility (Shang et al. 2019; González-Loyola and Petrova 2021). Also, aging can cause cervical lymph node atrophy and thickening of dorsal/ventral lymphatic structures, thus impacting the lymphatic output of the aged brain (Albayram et al. 2022). In MLVs specifically, such age-related dysfunctions reduce influx and outflux of CSF. It triggers the accumulation of metabolic products, accelerates neuroinflammation, induces the release of pro-inflammatory cytokines in the brain, and consequently aggravates cognitive dysfunction (Guo et al. 2023).

## Common features of neurodegenerative diseases in terms of the brain lymphatic system

Neurodegenerative diseases are characterized by a progressive decline in cognitive function following their initial onset, highlighting the need for an objective diagnosis rather than clinical assessments alone. From a metabolic and immunological perspective, common features can be observed in understanding the development of neurodegenerative diseases, such as the generation of unwanted protein plaques and the presence of contaminants measured within the brain. The accumulation of protein plaques, such as the tau neurofibrillary tangles seen in AD, can induce inflammation, synaptic impairment, and loss of neural function, resulting in the hallmark symptoms of cognitive and behavioral dysfunction associated with neurodegenerative diseases (Roda et al. 2022).

As described in the preceding section, aging has a direct impact on glymphatic and lymphatic functions, impairing the functionality of brain lymphatics. The accumulation of proteins in neurodegenerative diseases is closely associated with the maintenance of the brain lymphatics, which is responsible for removing soluble contaminants within the brain. Therefore, changes or disruptions in CSF drainage leading to impaired glymphatic/lymphatic function, have been observed to be correlated with the accumulation of specific pathological proteins known to be associated with neurodegenerative diseases. As the glymphatic system is responsible for the clearance of parenchymal metabolic wastes, it has been considered to be mainly involved in the pathogenesis of proteinopathies of neurodegenerative diseases such as AD or PD (Buccellato et al. 2022). PET study with taupathy tracer has shown that there is CSF clearance abnormality in lateral ventricle and superior nasal turbinate of AD patients, implying that decrease in ventricular CSF clearance is related with increased Amyloid beta (Aβ) deposition of AD (de Leon et al. 2017). Moreover, in experiment done on AD model transgenic mouse, functional disruption of MLVs induced deposition of Aβ and accelerated parenchymal Aβ accumulation, consistent with meningeal pathology in human AD. This indicates that meningeal lymphatic dysfunction might also be one of the major aggravating factors in AD pathology (Da Mesquita et al. 2018b).

## New perspectives for monitoring neurodegenerative diseases based on the CSF drainage route into the nasal lymphatics

Since CSF is encompasses the metabolic-pathological profile of the CNS, monitoring of CSF has emerged as a primary tool for identifying biomarkers essential diagnosing of neurological symptoms, including neurodegenerative diseases (Anoop et al. 2010). Key findings of CSF outflow relations in various pathological conditions of CNS are summarized in Table 1.

**Table 1.** CSF outflow relations in pathological conditions of CNS.

Pathological conditions in CNS	CSF outflow relations	Details	Reference
CSF abnormality	Outflow deficit.	CSF outflow deficit engage in the pathogenesis of hydrocephalus	(Krishnamurthy and Li 2014; Sokołowski et al. 2018)
Tumor development	Outflow deficit. Outflow alteration.	CSF outflow decrease in brain tumor development.	(Ma et al. 2019; Jiang et al. 2022)
Mental disorder	Outflow alteration.	Major schizophrenia features induced by CSF signaling	(Bechter 2013)
Strokes	Outflow alteration. Composition alteration.	Abnormal CSF flow inducing pathological changes. Change in iron concentration differentiates hemorrhage and ischemic strokes	(García-Cabo et al. 2020; Fang et al. 2022)
Neurodegenerative symptoms	Outflow alteration. Composition alteration.	Glymphatic malfunction in proteinopathy of late-onset neurodegenerative diseases like Alzheimer’s disease, Parkinson’s disease, Huntington’s disease or motor neuron diseases like multiple sclerosis.	(Ethell 2014; Bezerra et al. 2018; Visanji et al. 2018; Kwon et al. 2019; Guo et al. 2020; Verghese et al. 2022; Liu et al. 2023)
Neurological disorders	Outflow alteration. Composition alteration.	Microparticles and exosomes secreted into CSF in lumbosacral pain, Glymphatic alterations, and hemolytic accumulation occurs in traumatic brain injury	(Bechter and Schmitz 2014; Hansen et al. 2017; Rasmussen et al. 2018)

To maintain homeostasis in the brain, the glymphatic system exists to remove parenchymal waste through CSF drainage into the lymphatic system. The main route for CSF lymphatic drainage is the path connecting the brain and the nose. Previous studies have visually confirmed this pathway and demonstrated several correlations between the brain and nasal regions that have either been observed or are speculated to occur through this route. This suggests a strong possibility that events in the brain, such as the onset of neurodegenerative diseases, can be reflected through this pathway. Indeed, the influence of the glymphatic pathway has been evidenced by the detection of CSF-related markers in extracranial sources for certain neurological disorders. For instance, though the use of established biomarkers for AD, changes have been observed in plasma, leading to the establishment of blood test technology (Lee et al. 2020a). In addition, beta-amyloid oligomers, which are prominent markers of AD, have been detected in nasal discharge, indicating their potential utility as markers of disease progression (Yoo et al. 2020).

Moreover, in some neurodegenerative diseases, abnormalities related to olfaction, such as early loss of olfactory function, have been observed, indicating a correlation between the onset of the disease and the olfactory system. Therefore, metabolic changes in brain might be reflected in the olfactory system via the CSF outflow path into the nasal lymphatics. This can provide a new perspective for the diagnosis and monitoring of neurodegenerative diseases. Previous studies have conducted an Alzheimer's disease biomarker study using nasal discharge, establishing a validated protocol for the collection and processing of nasal samples. Samples obtained from the nasal roof adjacent to the olfactory bulb, subjected to proper sonication and centrifugation, have been identified as a source for assessing the pathological state of neurodegenerative diseases like Alzheimer's disease through analytical approaches such as proteomics (Kim et al. 2019; Yoo et al. 2020).

## Concluding remarks

We have reviewed the overall process of CSF circulation within the brain and its route of drainage into extracranial lymphatics and particularly focused on the pathway through the cribriform plate. During this exploration, we have described how the existence of this route has been documented and interpreted in previous literature, highlighting the interconnected changes between the brain and areas beyond the cribriform plate. Furthermore, we have discussed the potential implications of these findings for the diagnosis and monitoring of neurodegenerative diseases.

CSF circulates within the CNS and contributes to the glymphatic system of brain lymphatics by draining contaminants outward into the extracranial lymphatics. The CSF outflow path through the cribriform plate is one of the major pathways of CSF drainage. As it passes through the cribriform plate, CSF follows the olfactory nerves and exits into the nasal mucosa, eventually reaching the nasal lymphatics. From there, it can travel through the lymphatic network and ultimately reach the cervical lymph vessels.

This physical connection, bounded by the cribriform plate, allows for reciprocal influences between the two distinct systems: the CNS and olfactory system located in the nasal region. Changes in the nasal environment, such as variations in external factors including air pollution or the introduction of intranasally administered drugs, can impact CSF turnover, ultimately influencing the metabolic state of the brain. Conversely, changes in the brain can be transmitted to the external environment, leading to direct alterations in the extracranial areas.

As the CSF outflow route is directed towards lymphatic vessels, many neurological conditions cause changes in CSF physiology, and these resultant changes provide potential therapeutic avenues for brain disorders, including hydrocephalus and neurodegenerative diseases. CSF outflow plays a pivotal role in purging harmful substances from the CNS, thus contributing to immune surveillance in the brain. The significance of the function of CSF outflow carries profound pathological implications, underscoring its potential as a basis for therapeutic strategies for CNS diseases.

Certain correlations have been confirmed through this link between the brain and nasal area that CSF flows through. The evidence from previous studies suggests changes in the external environment affect the olfactory system and can be reflected in the CSF. Conversely, changes in CSF might be reflected in the extracranial nasal lymphatics and olfactory system.

Representing a significant risk factor in the progression of neurodegenerative diseases, aging is a major factor that induces critical changes in CSF turnover. CSF turnover alterations caused by aging or neurodegenerative diseases have already been well demonstrated. It can be inferred that changes in extracranial areas, including the olfactory system, may also be influenced by CSF outflow changes, as observed in the onset of neurodegenerative diseases. Olfactory dysfunction is one of the early symptoms in several neurodegenerative diseases and also suggests pathological relationships with the olfactory region in these conditions. Previous research has already demonstrated that, given the existence of a drainage route from the brain to the nasal mucosa, nasal exudate samples potentially serve as a new source of biomarkers for CNS diseases (García-Cabo et al. 2020). Therefore, considering this pathological link between the brain and nasal area provided by certain outflow paths of CSF might suggest a novel, non-invasive tool for the diagnosis and monitoring of neurodegenerative diseases.

## References

[CIT0001] Albayram MS, Smith G, Tufan F, Tuna IS, Bostancıklıoğlu M, Zile M, Albayram O. 2022. Non-invasive MR imaging of human brain lymphatic networks with connections to cervical lymph nodes. Nat Commun. 13(1):203. doi:10.1038/s41467-021-27887-0. 35017525 PMC8752739

[CIT0002] Anoop A, Singh PK, Jacob RS, Maji SK. 2010. Csf biomarkers for Alzheimer's disease diagnosis. Int J Alzheimers Dis. 2010:Article id 606802. doi:10.4061/2010/606802. PMC2915796 20721349

[CIT0003] Aspelund A, Antila S, Proulx ST, Karlsen TV, Karaman S, Detmar M, Wiig H, Alitalo K. 2015. A dural lymphatic vascular system that drains brain interstitial fluid and macromolecules. J Exp Med. 212(7):991–999. doi:10.1084/jem.20142290. 26077718 PMC4493418

[CIT0004] Bechter K. 2013. Updating the mild encephalitis hypothesis of schizophrenia. Prog Neuropsychopharmacol Biol Psychiatry. 42:71–91. doi:10.1016/j.pnpbp.2012.06.019. 22765923

[CIT0005] Bechter K, Schmitz B. 2014. Cerebrospinal fluid outflow along lumbar nerves and possible relevance for pain research: case report and review. Croat Med J. 55(4):399–404. doi:10.3325/cmj.2014.55.399. 25165054 PMC4157386

[CIT0006] Bezerra MLS, Ferreira ACAF, de Oliveira-Souza R. 2018. Pseudotumor cerebri and glymphatic dysfunction. Front Neurol. 8:734. doi:10.3389/fneur.2017.00734. 29387036 PMC5775972

[CIT0007] Boulton M, Armstrong D, Flessner M, Hay J, Szalai JP, Johnston M. 1998. Raised intracranial pressure increases CSF drainage through arachnoid villi and extracranial lymphatics. Am J Physiol. 275(3):R889–R896. doi:10.1152/ajpregu.1998.275.3.R889. 9728088

[CIT0008] Bradbury MW, Westrop RJ. 1983. Factors influencing exit of substances from cerebrospinal fluid into deep cervical lymph of the rabbit. J Physiol. 339:519–534. doi:10.1113/jphysiol.1983.sp014731. 6411905 PMC1199176

[CIT0009] Brady M, Rahman A, Combs A, Venkatraman C, Kasper RT, McQuaid C, Kwok WE, Wood RW, Deane R. 2020. Cerebrospinal fluid drainage kinetics across the cribriform plate are reduced with aging. Fluids Barriers CNS. 17(1):71. doi:10.1186/s12987-020-00233-0. 33256800 PMC7706057

[CIT0010] Buccellato FR, D'Anca M, Serpente M, Arighi A, Galimberti D. 2022. The role of glymphatic system in Alzheimer's and Parkinson's disease pathogenesis. Biomedicines. 10(9):2261. doi:10.3390/biomedicines10092261. 36140362 PMC9496080

[CIT0011] Da Mesquita S, Fu Z, Kipnis J. 2018a. The meningeal lymphatic system: A New player in neurophysiology. Neuron. 100(2):375–388. doi:10.1016/j.neuron.2018.09.022. 30359603 PMC6268162

[CIT0012] Da Mesquita S, Louveau A, Vaccari A, Smirnov I, Cornelison RC, Kingsmore KM, Contarino C, Onengut-Gumuscu S, Farber E, Raper D, et al. 2018b. Functional aspects of meningeal lymphatics in ageing and Alzheimer's disease. Nature. 560(7717):185–191. doi:10.1038/s41586-018-0368-8. 30046111 PMC6085146

[CIT0013] Dan X, Wechter N, Gray S, Mohanty JG, Croteau DL, Bohr VA. 2021. Olfactory dysfunction in aging and neurodegenerative diseases. Ageing Res Rev. 70:101416. doi:10.1016/j.arr.2021.101416. 34325072 PMC8373788

[CIT0014] de Leon MJ, Li Y, Okamura N, Tsui WH, Saint-Louis LA, Glodzik L, Osorio RS, Fortea J, Butler T, Pirraglia E, et al. 2017. Cerebrospinal fluid clearance in Alzheimer disease measured with dynamic PET. J Nucl Med. 58(9):1471–1476. doi:10.2967/jnumed.116.187211. 28302766 PMC5577629

[CIT0015] Engelhardt B, Vajkoczy P, Weller RO. 2017. The movers and shapers in immune privilege of the CNS. Nat Immunol. 18(2):123–131. doi:10.1038/ni.3666. 28092374

[CIT0016] Ethell DW. 2014. Disruption of cerebrospinal fluid flow through the olfactory system may contribute to Alzheimer's disease pathogenesis. J Alzheimers Dis. 41(4):1021–1030. doi:10.3233/JAD-130659. 24769627

[CIT0017] Faber WM. 1937. The nasal mucosa and the subarachnoid space. Am J Anat 62:121–148. doi:10.1002/aja.1000620106.

[CIT0018] Fang Y, Huang L, Wang X, Si X, Lenahan C, Shi H, Shao A, Tang J, Chen S, Zhang J, Zhang JH. 2022. A new perspective on cerebrospinal fluid dynamics after subarachnoid hemorrhage: from normal physiology to pathophysiological changes. J Cereb Blood Flow Metab. 42(4):543–558. doi:10.1177/0271678X211045748. 34806932 PMC9051143

[CIT0019] Furukawa M, Shimoda H, Kajiwara T, Kato S, Yanagisawa S. 2008. Topographic study on nerve-associated lymphatic vessels in the murine craniofacial region by immunohistochemistry and electron microscopy. Biomed Res. 29(6):289–296. doi:10.2220/biomedres.29.289. 19129672

[CIT0020] García-Cabo C, Llano-Suárez P, Benavente-Fernández L, Calleja-Puerta S, Costa-Fernández JM, Fernández-Abedul MT. 2020. Obtaining information from the brain in a non-invasive way: determination of iron in nasal exudate to differentiate hemorrhagic and ischemic strokes. Clin Chem Lab Med. 58(5):847–853. doi:10.1515/cclm-2019-0899. 31730519

[CIT0021] González-Loyola A, Petrova TV. 2021. Development and aging of the lymphatic vascular system. Adv Drug Deliv Rev. 169:63–78. doi:10.1016/j.addr.2020.12.005. 33316347

[CIT0022] Guo P, Wang RD, Lian TH, Ding DY, Zhang YN, Zhang WJ, Li DN, Li LX, Li JH, Guan HY, et al. 2020. Olfactory dysfunction and Its association With neuropathologic proteins in cerebrospinal fluid from patients With Parkinson disease. Front Aging Neurosci. 12:594324. doi:10.3389/fnagi.2020.594324. 33362530 PMC7759606

[CIT0023] Guo X, Zhang G, Peng Q, Huang L, Zhang Z, Zhang Z. 2023. Emerging roles of meningeal lymphatic vessels in Alzheimer's disease. J Alzheimers Dis. 94(s1):S355–S366. doi:10.3233/JAD-221016. 36683509 PMC10473149

[CIT0024] Hablitz LM, Nedergaard M. 2021. The glymphatic system: A novel component of fundamental neurobiology. J Neurosci. 41(37):7698–7711. doi:10.1523/JNEUROSCI.0619-21.2021. 34526407 PMC8603752

[CIT0025] Hablitz LM, Plá V, Giannetto M, Vinitsky HS, Stæger FF, Metcalfe T, Nguyen R, Benrais A, Nedergaard M. 2020. Circadian control of brain glymphatic and lymphatic fluid flow. Nat Commun. 11(1):4411. doi:10.1038/s41467-020-18115-2. 32879313 PMC7468152

[CIT0026] Hansen EA, Romanova L, Janson C, Lam CH. 2017 Jun. The effects of blood and blood products on the arachnoid cell. Exp Brain Res. 235(6):1749–1758. doi:10.1007/s00221-017-4927-2. 28285405

[CIT0027] Hladky SB, Barrand MA. 2022. The glymphatic hypothesis: the theory and the evidence. Fluids Barriers CNS. 19(1):9. 35115036 10.1186/s12987-021-00282-z PMC8815211

[CIT0028] Hsu M, Laaker C, Madrid A, et al. 2022. Neuroinflammation creates an immune regulatory niche at the meningeal lymphatic vasculature near the cribriform plate. Nat Immunol. 23:581–593. doi:10.1038/s41590-022-01158-6. 35347285 PMC8989656

[CIT0029] Hsu M, Rayasam A, Kijak JA, Choi YH, Harding JS, Marcus SA, Karpus WJ, Sandor M, Fabry Z. 2019. Neuroinflammation-induced lymphangiogenesis near the cribriform plate contributes to drainage of CNS-derived antigens and immune cells. Nat Commun. 10(1):229. doi:10.1038/s41467-018-08163-0. 30651548 PMC6335416

[CIT0030] Hsu M, Sandor M, Fabry Z. 2021. Current concepts on communication between the central nervous system and peripheral immunity via lymphatics: what roles do lymphatics play in brain and spinal cord disease pathogenesis?. Biol Futur. 72(1):45–60. doi:10.1007/s42977-021-00066-4. 34554497

[CIT0031] Hu Y, Ding WT, Zhu XN, Wang XL. 2015. A mini review: Tau transgenic mouse models and olfactory dysfunction in Alzheimer's disease. Zhongguo Ying Yong Sheng Li Xue Za Zhi. 31(6):481–490. 27215014

[CIT0032] Iliff JJ, Wang M, Liao Y, Plogg BA, Peng W, Gundersen GA, Benveniste H, Vates GE, Deane R, Goldman SA, et al. 2012. A paravascular pathway facilitates CSF flow through the sbrain parenchyma and the clearance of interstitial solutes, including amyloid β. Sci Transl Med. 4(147):147ra111. doi:10.1126/scitranslmed.3003748. PMC3551275 22896675

[CIT0033] Illes S. 2017. More than a drainage fluid: the role of CSF in signaling in the brain and other effects on brain tissue. Handb Clin Neurol. 146:33–46. doi:10.1016/B978-0-12-804279-3.00003-4. 29110778

[CIT0034] Jacob L, de Brito Neto J, Lenck S, Corcy C, Benbelkacem F, Geraldo LH, Xu Y, Thomas JM, El Kamouh MR, Spajer M, et al. 2022. Conserved meningeal lymphatic drainage circuits in mice and humans. J Exp Med. 219(8):e20220035. doi:10.1084/jem.20220035. 35776089 PMC9253621

[CIT0035] Jakic B, Kerjaschki D, Wick G. 2020. Lymphatic capillaries in aging. Gerontology. 66(5):419–426. doi:10.1159/000508459. 32580201

[CIT0036] Jiang H, Wei H, Zhou Y, Xiao X, Zhou C, Ji X. 2022. Overview of the meningeal lymphatic vessels in aging and central nervous system disorders. Cell Biosci. 12(1):202. doi:10.1186/s13578-022-00942-z. 36528776 PMC9759913

[CIT0037] Johanson CE, Duncan JA, Klinge PM, Brinker T, Stopa EG, Silverberg GD. 2008. Multiplicity of cerebrospinal fluid functions: New challenges in health and disease. Cerebrospinal Fluid Res. 5:10. doi:10.1186/1743-8454-5-10. 18479516 PMC2412840

[CIT0038] Johnston M, Zakharov A, Papaiconomou C, Salmasi G, Armstrong D. 2004. Evidence of connections between cerebrospinal fluid and nasal lymphatic vessels in humans, non-human primates and other mammalian species. Cerebrospinal Fluid Res. 1(1):2. doi:10.1186/1743-8454-1-2. 15679948 PMC546409

[CIT0039] Kida S, Pantazis A, Weller RO. 1993. CSF drains directly from the subarachnoid space into nasal lymphatics in the rat. anatomy, histology and immunological significance. Neuropathol Appl Neurobiol. 19(6):480–488. doi:10.1111/j.1365-2990.1993.tb00476.x. 7510047

[CIT0040] Kim H, Moore SA, Johnston MG. 2014. Potential for intranasal drug delivery to alter cerebrospinal fluid outflow via the nasal turbinate lymphatics. Fluids Barriers CNS. 11(1):4. doi:10.1186/2045-8118-11-4. 24528926 PMC3927830

[CIT0041] Kim JY, Rasheed A, Yoo SJ, Kim SY, Cho B, Son G, Yu SW, Chang KA, Suh YH, Moon C. 2018. Distinct amyloid precursor protein processing machineries of the olfactory system. Biochem Biophys Res Commun. 495(1):533–538. doi:10.1016/j.bbrc.2017.10.153. 29097202

[CIT0042] Kim YH, Lee SM, Cho S, Kang JH, Minn YK, Park H, Choi SH. 2019. Amyloid beta in nasal secretions may be a potential biomarker of Alzheimer's disease. Sci Rep. 9(1):4966. doi:10.1038/s41598-019-41429-1. 30899050 PMC6428828

[CIT0043] Krishnamurthy S, Li J. 2014. New concepts in the pathogenesis of hydrocephalus. Transl Pediatr. 3(3):185–194. doi:10.3978/j.issn.2224-4336.2014.07.02. 26835336 PMC4729848

[CIT0044] Kwon S, Moreno-Gonzalez I, Taylor-Presse K, Iii E, Gamez G, Calderon N, Zhu O, Velasquez B, Soto FC, Sevick-Muraca C, M E. 2019. Impaired peripheral lymphatic function and cerebrospinal fluid outflow in a mouse model of Alzheimer's disease. J Alzheimers Dis. 69(2):585–593. doi:10.3233/JAD-190013. 31104026 PMC7891904

[CIT0045] Lee H, Ugay D, Hong S, Kim Y. 2020a. Alzheimer's disease diagnosis using misfolding proteins in blood. Dement Neurocogn Disord. 19(1):1–18. doi:10.12779/dnd.2020.19.1.1. 32174051 PMC7105719

[CIT0046] Lee Y, Choi Y, Park EJ, Kwon S, Kim H, Lee JY, Lee DS. 2020b. Improvement of glymphatic-lymphatic drainage of beta-amyloid by focused ultrasound in Alzheimer's disease model. Sci Rep. 10(1):16144. doi:10.1038/s41598-020-73151-8. 32999351 PMC7527457

[CIT0047] Lehman M, Silver R. 2000. CSF signaling in physiology and behavior. Prog Brain Res. 125:415–433. doi:10.1016/S0079-6123(00)25029-2. 11098676

[CIT0048] Li G, Cao Y, Tang X, Huang J, Cai L, Zhou L. 2022. The meningeal lymphatic vessels and the glymphatic system: potential therapeutic targets in neurological disorders. J Cereb Blood Flow Metab. 42(8):1364–1382. doi:10.1177/0271678X221098145. 35484910 PMC9274866

[CIT0049] Liu H, Ni Z, Chen Y, Wang D, Qi Y, Zhang Q, Wang S. 2012. Olfactory route for cerebrospinal fluid drainage into the cervical lymphatic system in a rabbit experimental model. Neural Regen Res. 7(10):766–771. doi:10.3969/j.issn.1673-5374.2012.10.009. 25737700 PMC4345659

[CIT0050] Liu Z, Huang Y, Wang X, Li JY, Zhang C, Yang Y, Zhang J. 2023. The cervical lymph node contributes to peripheral inflammation related to Parkinson's disease. J Neuroinflammation. 20(1):93. doi:10.1186/s12974-023-02770-5. 37038192 PMC10088204

[CIT0051] Louveau A, Plog BA, Antila S, Alitalo K, Nedergaard M, Kipnis J. 2017. Understanding the functions and relationships of the glymphatic system and meningeal lymphatics. J Clin Invest. 127(9):3210–3219. doi:10.1172/JCI90603. 28862640 PMC5669566

[CIT0052] Louveau A, Smirnov I, Keyes TJ, Eccles JD, Rouhani SJ, Peske JD, Derecki NC, Castle D, Mandell JW, Lee KS, et al. 2015. Structural and functional features of central nervous system lymphatic vessels. Nature. 523(7560):337–341. doi:10.1038/nature14432. 26030524 PMC4506234

[CIT0053] Ma Q, Ineichen BV, Detmar M, et al. 2017. Outflow of cerebrospinal fluid is predominantly through lymphatic vessels and is reduced in aged mice. Nat Commun. 8:1434. doi:10.1038/s41467-017-01484-6. 29127332 PMC5681558

[CIT0054] Ma Q, Schlegel F, Bachmann SB, Schneider H, Decker Y, Rudin M, Weller M, Proulx ST, Detmar M. 2019. Lymphatic outflow of cerebrospinal fluid is reduced in glioma. Sci Rep. 9(1):14815. doi:10.1038/s41598-019-51373-9. 31616011 PMC6794292

[CIT0055] Mehta NH, Sherbansky J, Kamer AR, Carare RO, Butler T, Rusinek H, Chiang GC, Li Y, Strauss S, Saint-Louis LA, et al. 2022. The brain-nose interface: A potential cerebrospinal fluid clearance site in humans. Front Physiol. 12:769948. doi:10.3389/fphys.2021.769948. 35058794 PMC8764168

[CIT0056] Nagra G, Koh L, Zakharov A, Armstrong D, Johnston M. 2006. Quantification of cerebrospinal fluid transport across the cribriform plate into lymphatics in rats. Am J Physiol Regul Integr Comp Physiol. 291(5):R1383–9. doi:10.1152/ajpregu.00235.2006. 16793937

[CIT0057] Nagra G, Koh L, Zakharov A, Armstrong D, Johnston M. 2006. Quantification of cerebrospinal fluid transport across the cribriform plate into lymphatics in rats. Am J Physiol Regul Integr Comp Physiol. 291(5):R1383–9. doi:10.1152/ajpregu.00235.2006. 16793937

[CIT0058] Noé FM, Marchi N. 2019. Central nervous system lymphatic unit, immunity, and epilepsy: Is there a link? Epilepsia Open. 4(1):30–39. doi:10.1002/epi4.12302. 30868113 PMC6398113

[CIT0059] Norwood JN, Zhang Q, Card D, Craine A, Ryan TM, Drew PJ. 2019. Anatomical basis and physiological role of cerebrospinal fluid transport through the murine cribriform plate. eLife. 8:e44278. doi:10.7554/eLife.44278. 31063132 PMC6524970

[CIT0060] Nycz B, Mandera M. 2021. The features of the glymphatic system. Auton Neurosci. 232:102774. doi:10.1016/j.autneu.2021.102774. 33610009

[CIT0061] Pedersen SH, Lilja-Cyron A, Andresen M, Juhler M. 2018. The relationship between intracranial pressure and age-chasing age-related reference values. World Neurosurg. 110:e119–e123. doi:10.1016/j.wneu.2017.10.086. 29107158

[CIT0062] Pedler MG, Petrash JM, Subramanian PS. 2021. Prostaglandin analog effects on cerebrospinal fluid reabsorption via nasal mucosa. PLoS One. 16(12):e0248545. doi:10.1371/journal.pone.0248545. 34971554 PMC8719688

[CIT0063] Persson NDÅ, Uusalo P, Nedergaard M, Lohela TJ, Lilius TO. 2022. Could dexmedetomidine be repurposed as a glymphatic enhancer? Trends Pharmacol Sci. 43(12):1030–1040. doi:10.1016/j.tips.2022.09.007. 36280451

[CIT0064] Proulx ST. 2021. Cerebrospinal fluid outflow: a review of the historical and contemporary evidence for arachnoid villi, perineural routes, and dural lymphatics. Cell Mol Life Sci. 78(6):2429–2457. doi:10.1007/s00018-020-03706-5. 33427948 PMC8004496

[CIT0065] Rasmussen JC, Kwon S, Pinal A, Bareis A, Velasquez FC, Janssen CF, Morrow JR, Fife CE, Karni RJ, Sevick-Muraca EM. 2020. Assessing lymphatic route of CSF outflow and peripheral lymphatic contractile activity during head-down tilt using near-infrared fluorescence imaging. Physiol Rep. 8(4):e14375. doi:10.14814/phy2.14375. 32097544 PMC7058174

[CIT0066] Rasmussen MK, Mestre H, Nedergaard M. 2018. The glymphatic pathway in neurological disorders. Lancet Neurol. 17(11):1016–1024. doi:10.1016/S1474-4422(18)30318-1. 30353860 PMC6261373

[CIT0067] Roda AR, Serra-Mir G, Montoliu-Gaya L, Tiessler L, Villegas S. 2022. Amyloid-beta peptide and tau protein crosstalk in Alzheimer's disease. Neural Regen Res. 17(8):1666–1674. doi:10.4103/1673-5374.332127. 35017413 PMC8820696

[CIT0068] Sakka L, Coll G, Chazal J. 2011 Dec. Anatomy and physiology of cerebrospinal fluid. Eur Ann Otorhinolaryngol Head Neck Dis. 128(6):309–316. doi:10.1016/j.anorl.2011.03.002. 22100360

[CIT0069] Shang T, Liang J, Kapron CM, Liu J. 2019. Pathophysiology of aged lymphatic vessels. Aging (Albany NY). 11(16):6602–6613. doi:10.18632/aging.102213. 31461408 PMC6738433

[CIT0070] Simon MJ, Iliff JJ. 2016. Regulation of cerebrospinal fluid (CSF) flow in neurodegenerative, neurovascular and neuroinflammatory disease. Biochim Biophys Acta. 1862(3):442–451. doi:10.1016/j.bbadis.2015.10.014. 26499397 PMC4755861

[CIT0071] Sokołowski W, Barszcz K, Kupczyńska M, Czubaj N, Skibniewski M, Purzyc H. 2018. Lymphatic drainage of cerebrospinal fluid in mammals - are arachnoid granulations the main route of cerebrospinal fluid outflow? Biologia (Bratisl). 73(6):563–568. doi:10.2478/s11756-018-0074-x. 30147112 PMC6097054

[CIT0072] Spera I, Cousin N, Ries M, Kedracka A, Castillo A, Aleandri S, Vladymyrov M, Mapunda JA, Engelhardt B, Luciani P, et al. 2023. Open pathways for cerebrospinal fluid outflow at the cribriform plate along the olfactory nerves. EBioMedicine. 91:104558. doi:10.1016/j.ebiom.2023.104558. 37043871 PMC10119713

[CIT0073] Sun BL, Wang LH, Yang T, Sun JY, Mao LL, Yang MF, Yuan H, Colvin RA, Yang XY. 2018. Lymphatic drainage system of the brain: A novel target for intervention of neurological diseases. Prog Neurobiol. 163:118–143. doi:10.1016/j.pneurobio.2017.08.007. 28903061

[CIT0074] Tamura R, Yoshida K, Toda M. 2020 Aug. Current understanding of lymphatic vessels in the central nervous system. Neurosurg Rev. 43(4):1055–1064. doi:10.1007/s10143-019-01133-0. 31209659

[CIT0075] Verghese JP, Terry A, de Natale ER, Politis M. 2022 Nov 25. Research evidence of the role of the glymphatic system and Its potential pharmacological modulation in neurodegenerative diseases. J Clin Med. 11(23):6964. doi:10.3390/jcm11236964. 36498538 PMC9735716

[CIT0076] Vinje V, Eklund A, Mardal KA, Rognes ME, Støverud KH. 2020. Intracranial pressure elevation alters CSF clearance pathways. Fluids Barriers CNS. 17(1):29. doi:10.1186/s12987-020-00189-1. 32299464 PMC7161287

[CIT0077] Visanji NP, Lang AE, Munoz DG. 2018. Lymphatic vasculature in human dural superior sagittal sinus: implications for neurodegenerative proteinopathies. Neurosci Lett. 665:18–21. doi:10.1016/j.neulet.2017.11.001. 29133178

[CIT0078] Weller RO, Sharp MM, Christodoulides M, Carare RO, Møllgård K. 2018. The meninges as barriers and facilitators for the movement of fluid, cells and pathogens related to the rodent and human CNS. Acta Neuropathol. 135(3):363–385. doi:10.1007/s00401-018-1809-z. 29368214

[CIT0079] Yankova G, Bogomyakova O, Tulupov A. 2021. The glymphatic system and meningeal lymphatics of the brain: new understanding of brain clearance. Rev Neurosci. 32(7):693–705. doi:10.1515/revneuro-2020-0106. 33618444

[CIT0080] Yoo SJ, Son G, Bae J, Kim SY, Yoo YK, Park D, Baek SY, Chang KA, Suh YH, Lee YB, et al. 2020. Longitudinal profiling of oligomeric Aβ in human nasal discharge reflecting cognitive decline in probable Alzheimer's disease. Sci Rep. 10(1):11234. doi:10.1038/s41598-020-68148-2. 32641719 PMC7343787

